# Inhibitory motoneurons in arthropod motor control: organisation, function, evolution

**DOI:** 10.1007/s00359-014-0922-2

**Published:** 2014-06-26

**Authors:** Harald Wolf

**Affiliations:** 1 Stellenbosch Institute for Advanced Study (STIAS), Wallenberg Research Centre at Stellenbosch University, 10 Marais Street, Stellenbosch, 7600 South Africa; 2 Institute of Neurobiology, University of Ulm, Ulm, Germany

**Keywords:** Arthropod, Motor control, Inhibitory motoneuron, Muscle fibre recruitment, Evolution

## Abstract

Miniaturisation of somatic cells in animals is limited, for reasons ranging from the accommodation of organelles to surface-to-volume ratio. Consequently, muscle and nerve cells vary in diameters by about two orders of magnitude, in animals covering 12 orders of magnitude in body mass. Small animals thus have to control their behaviour with few muscle fibres and neurons. Hexapod leg muscles, for instance, may consist of a single to a few 100 fibres, and they are controlled by one to, rarely, 19 motoneurons. A typical mammal has thousands of fibres per muscle supplied by hundreds of motoneurons for comparable behavioural performances. Arthopods—crustaceans, hexapods, spiders, and their kin—are on average much smaller than vertebrates, and they possess inhibitory motoneurons for a motor control strategy that allows a broad performance spectrum despite necessarily small cell numbers. This arthropod motor control strategy is reviewed from functional and evolutionary perspectives and its components are described with a focus on inhibitory motoneurons. Inhibitory motoneurons are particularly interesting for a number of reasons: evolutionary and phylogenetic comparison of functional specialisations, evolutionary and developmental origin and diversification, and muscle fibre recruitment strategies.

## Introduction

The central nervous systems of animals supply the muscles of body and limbs via motor nerves. The motor neurons in these nerves activate the muscles to produce contractions and ultimately control behaviour. Muscle relaxation occurs spontaneously after excitatory input has ceased, affording no particular control of muscle inactivation. Excitatory motoneurons are all that is needed, and they are the only motoneuron type present in vertebrates. This makes the existence of inhibitory motoneurons in arthropods appear enigmatic, and inhibitory motoneurons actually remain unknown even to many contemporary physiologists. By the same token, the function of inhibitory motoneurons has evaded proper understanding (Pearson and Bergmann 1969) for decades after their initial discovery (Wiersma 1941).

Nonetheless, these motoneurons are essential elements of the motor control strategy in malacostracan crustaceans, such as crabs, lobsters, and spiny lobsters, in hexapods (insects), and in spiders and their kin, the chelicerates. The ultimate reason for the evolution of inhibitory motoneurons probably lies in the small size of most arthropods, compared to vertebrates, resulting in a roughly proportionately small number of muscle and (motor) neurons. Small cell numbers cause problems for the smooth control of movement and for a broad spectrum of muscle performance, from tonic posture control to fast evasive movements or predatory strikes. In conjunction with a set of further features, inhibitory motoneurons allow the sophisticated control of muscle contraction with very few motoneurons. In this way, arthropods achieve motor performances that are quite comparable to those of higher vertebrates, a surprising feat considering their small bodies, small cell numbers, and other features, such as lack of axon myelination (reviews in Müller et al. 1992; Rathmayer 1990, 1997). Several invertebrate groups have, however, evolved structures equivalent to vertebrate myelination (review in Hartline and Colman 2007).

The study of inhibitory motor control in arthropods is interesting for a number of reasons. First, inhibitory motoneurons may provide suitable characters to resolve evolutionary questions (Kutsch and Breidbach 1994; Harzsch et al. 2005). This is because the complement and basic properties of inhibitory motoneurons appear to be conserved among different arthropod groups, while there are intriguing functional specialisations in detail that lend themselves for comparison. Second, good accessibility of arthropod central nervous and neuromuscular systems allows the study of inhibitory mechanisms in cellular and physiological detail and functional context (Atwood and Tse 1993; Rathmayer and Djokai 2000; Clarac and Pearlstein 2007). Third, aspects of arthropod (inhibitory) motor control may be informative for movement control from more general perspectives. This includes problems of allometry and cell numbers available for a given task such as muscle activation.

## The complement of inhibitory motoneurons supplying the arthropod appendage

Inhibitory motoneurons have been studied mainly in orthopteran hexapods and malacostracan crustaceans, covering virtually all aspects of peripheral inhibition from motor control aspects to cellular and molecular detail (reviews in Wiens 1989; Atwood and Tse 1993; Rathmayer 1997; Clarac and Pearlstein 2007). An outline of the complement of inhibitory motoneurons identified in these arthropod groups shall be given before addressing the principles of inhibitory motoneuron function. And the apparently ubiquitous presence of inhibitory motoneurons in arthropods shall be indicated as it emerges from the sparsely scattered studies in arthropods other than orthopterans and malacostracans.

**Fig. 1:**
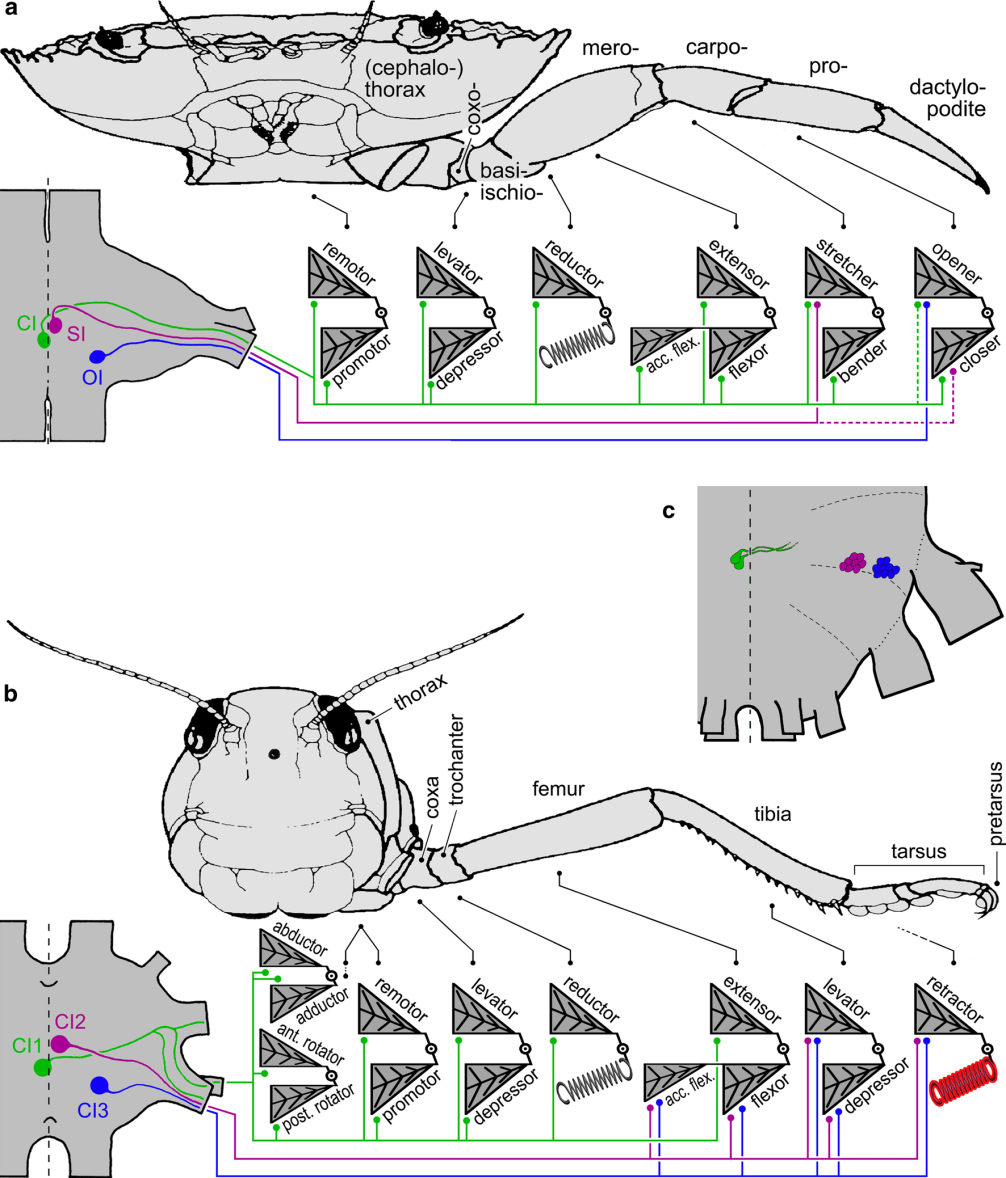
Comparison of inhibitory motoneuron supplies in, **a** decapod crustacean, **b** hexapod walking legs and scorpion inhibitory motoneurons (**c**). Thorax and walking leg of crab (**a)** and locust (**b**) are outlined at the *top*, respectively, with leg articles labelled. The corresponding pairs of antagonistic muscles are indicated *below* [dactylopodite (**a)** and pretarsus (**b)**, contain no muscles; *spring* symbols indicate elastic cuticle elements that work against muscles without antagonists; see also Fig. 6]. Inhibitory motoneuron supply is indicated in colours (*green*, common inhibitor and CI1; *violet*, stretcher inhibitor and CI2; *blue*, opener inhibitor and CI3; inhibitory synapses, *dots*). Similar colours indicate tentative homology (Wiens and Wolf 1993). Morphologies of inhibitory motoneurons in segmental ganglia are indicated in *ganglion diagrams*, *bottom* *left*, respectively [crayfish ganglion is shown for clarity in **a**, although the crab outlined above has a fused ventral nerve cord; however, neuromere structure in the crab corresponds closely to that of unfused ganglia in the crayfish (Wiens 1985, 1991, 1993; Homberg et al. 1993)]. Minor differences between the innervation patterns shown in **a** and Fig. 6 are due to the fact that Fig. 6 is a summary of connections observed in different species (Faulkes and Paul 1997), while **a** focuses on possibly vestigial innervation patterns (indicated by *dotted lines*; Cooke and Macmillan 1983; Wiens 1985, 1991, 1993). The posterior end of a (fused) subesophageal ganglion mass in a scorpion is shown in **c**. Inhibitory motoneuron somata supplying the 3rd walking leg are indicated in their corresponding neuromere; colours as in **a** and **b** to indicate possible homologies, although the exact innervation patterns are unknown (after Wolf and Harzsch 2002b)

Three inhibitory motoneurons exist for the walking legs of both malacostracans and hexapods (Wiens and Wolf 1993) (Fig. 1). A so-called **c**ommon **i**nhibitory motoneuron (abbreviation CI or CI neuron) supplies the majority of leg muscles in the orthopteran hexapods examined to date. This has been demonstrated in varying detail in locusts (Hale and Burrows 1985; Burrows 1996), stick insects (Bässler 1983), crickets (Böser 1999) and cockroaches (Pearson and Bergmann 1969). This inhibitor is termed CI1 (Fig. 1b). A common inhibitor with similar features supplies all leg muscles in the studied crab species (Wiens 1985 Wiens and Rathmayer 1985; Rathmayer and Bévengut 1986; see also; Ferrero and Wales 1976; Cooke and Macmillan 1983) and is termed CI (Fig. 1a). These two common inhibitors appear to be homologous according to available data. Characteristics used to judge homology include neuroanatomical features (Homberg et al. 1993), especially soma location, course of neurites, and arborisation areas with respect to landmarks in ganglion anatomy, nerve roots of exit, and muscle innervation pattern, including intriguing patterns of vestigial innervation (Wiens 1989; Wiens and Wolf 1993). This vestigial, or reduced and often dysfunctional, nerve supply may be indicative of the course of evolutionary change. The remaining two inhibitory motoneurons in the locust, CI2 and CI3, together supply a set of distal leg muscles (Hale and Burrows 1985) (Fig. 1b). In malacostracans, each of the two remaining inhibitors innervates just a single leg muscle, both thus representing specific inhibitors (Fig. 1a). One of the two is termed stretcher inhibitor, SI, the other opener inhibitor, OI, according to the muscles they innervate (Bévengut and Cournil 1990) (Fig. 1a). The topic of specific inhibitors shall be addressed further below. These two pairs of inhibitory motoneurons, too, appear to be homologous between hexapods and malacostracans according to the above criteria. Namely, CI2 and SI as well as CI3 and OI appear to be homologous neurons (Wiens and Wolf 1993). It would thus seem that a set of three inhibitory motoneurons per appendage is a common feature of hexapod and malacostracan walking legs, which may be interpreted as a plesiomorphic character set, or symplesiomorphy (Kutsch and Breidbach 1994; Harzsch et al. 2005).

Of course, the pattern of inhibitory motoneuron supply has been modified in the course of evolution. The mandible, for example, is a highly specialised appendage with a reduced number of segments (or, more correctly, articles, with the term segment reserved for body segments) and correspondingly reduced muscle supply, and it is not used for postural maintenance and locomotion but for chewing (Snodgrass 1950; Manton and Harding 1964). In this respect, its movements are more related to stomach, heart or ventilation movements, affording relatively constant but repetitive and long-lasting action. Since common inhibitory innervation serves to adjust muscle performance to a broad spectrum of movement speeds, one may expect that inhibitory innervation is absent in the mandible. This would correspond to the lack of inhibitory innervation in the ventilatory scaphognathite and stomach muscles to be addressed below. The hexapod mandible is apparently without inhibitory motoneuron supply, suggesting that all three limb muscle inhibitors have been lost in the course of evolution. The malacostracan mandible, by contrast, does have an inhibitory motoneuron (Ferrero and Wales 1976; Wales and Ferrero 1976). This may be interpreted as a reduced state of inhibitory motoneuron supply due to the specialised mandible function. One would expect that the remaining inhibitor corresponds to CI1 since it supplies the base of the appendage, an assumption that has however not been examined so far.

Similar and presumably reduced sets of inhibitory motoneurons were reported for other appendages that are not used for walking locomotion. The cricket and stick insect antenna, for instance, is supplied by a single common inhibitory motoneuron (Allgäuer and Honegger 1993; Dürr et al. 2001). The fact that there is only one inhibitory motoneuron may not be surprising since just the two basal antenna articles are moved by muscles, namely scape and pedicel. And inhibitory innervation *per se* makes good sense in view of the rapid pointing movements performed towards visual targets in crickets (Honegger 1981; Yamawaki and Ishibashi 2014) and the fast exploratory and searching movements of the antennae observed in walking stick insects (Dürr et al. 2001). Possible functions of the single inhibitory motoneuron in the crayfish uropod (Nagayama 1999) have not yet been addressed. Enabling rapid expansion of the tail fan in the context of tail flip or swimming behaviour is a distinct possibility here.

It has long remained enigmatic why a set of motoneurons neurons or even a single (inhibitory) motoneuron should supply most or all muscles of a leg (Wiersma 1941; Cooke and Macmillan 1983; Rathmayer and Bévengut 1986) (Fig. 1), thus apparently serving some global function independent of the control of the contraction of an individual muscle. Only the detailed understanding arthropod neuromuscular organisation and that it differs substantially from the better studied vertebrate muscle has eventually revealed the function of common inhibitory motoneurons.

## Motor control in small animals: both nerve and muscle cells are of roughly similar sizes throughout the animal kingdom

The cellular characteristics of muscle fibres are notably similar throughout the animal kingdom. This is particularly striking when comparing the well-studied skeletal muscles of vertebrates and arthropods, and it indicates that specialised muscle cells already existed before these major animal groups diverged (Seipel and Schmid 2005). Important for the present purpose is the fact that even the sizes of muscle cells remain within a relatively narrow range. The masses of muscle cells vary by not quite five orders of magnitude, even when considering developmental change—which is not appropriate here since developing muscle cells are not yet functional—and extreme specialisation (Eisenberg 1983; Dudel et al. 2001); the masses of most muscle cells thus range within 3 orders of magnitude. By contrast, the organisms propelled by these muscle cells cover 12 orders of magnitude in body mass. What really counts for muscle cells is their cross-sectional area that produces force by means of the actin and myosin filaments accommodated in relatively constant molecular arrangement in skeletal muscle. This holds true despite specialisations of muscle fibres for different functions, most notably fast contracting and slow contracting fibre populations (Rathmayer and Maier 1987). Cross-sectional area varies by much less, naturally, by just about two orders of magnitude. More commonly published are fibre diameters, ranging from 5 µm in miniaturized ptiliid beetles (Grebennikov and Beutel 2002) through 25 micrometers for the fruit fly, a more typical value for hexapods, to 10–80 µm in mammals including the biggest whales (Eisenberg 1983). Notably, differences in muscle fibre size appear to be related more to function (Rathmayer and Maier 1987) than to animal species. The same line of argument holds for neurons, at least as long as they are not myelinated. Even myelinated nerve axons follow the same rules but are just about an order of magnitude smaller (Hartline and Colman 2007).

The relative constancy of muscle fibre size, and particularly fibre diameter, is due to the fact that muscle cells cannot be miniaturized or enlarged indefinitely according to allometric scaling laws (overview in Schmidt-Nielsen 2002). These limitations are particularly strict for metabolically active tissue like muscle (review in Wieser 1995). Limiting factors for cell enlargement include the requirements of nutrient supply through transport networks of the cytoskeleton or through diffusion within the cell volume (compare West et al. 2002). Nutrient supply is also limited by diffusion through the cell membrane which encloses a cell volume increasing disproportionately with cell diameter. Cell volume increases roughly to the third power with cell diameter, and membrane area roughly to the second power, which results in the surface-to-volume ratio becoming less favourable for membrane transport with a power of 2/3. Cell miniaturization is restricted by, among other factors, the limited power density of (oxidative) energy delivery systems. Namely, the membrane-bound respiratory chain prevents reductions in mitochondrion size, and thus also in cell size, below a certain limit (review in Wieser 1995). The relatively low ion content of excitable cells with a high surface-to-volume ratio, and the small number of ion channels in the membranes of small cells are other limiting factors. In axons below about 0.5–0.1 µm in diameter, noise from ion channel switching and the loss of ions through the cell membrane during an action potential compromise spike signalling (Laughlin and Sejnowski 2003; Faisal et al. 2005). These lines of argument are strictly true only for cells with a constant geometry of their shape, while muscle and nerve cells tend to become longer in larger animals to bridge larger distances, with their diameters changing little or not at all. This tends to exacerbate the above problem, in principle, although it becomes irrelevant when comparing organisms across 12 orders of magnitude in body mass.

## Small animals have to control movement with fewer muscle and nerve cells

The roughly similar diameter ranges of axons as well as muscle fibres throughout the animal kingdom—with the notable exception of myelinated vertebrate axons—have the important consequence that small animals have to live with much smaller numbers of nerve and muscle cells. While small cell numbers are quite irrelevant in the case of metabolic tissues, control problems arise in the cases of nerve and muscle cells. These are motor control problems in the sense that fewer motor units, the groups of muscle fibres supplied by a given motoneuron, are available for the smooth grading of force output and for the selection of tonic and phasic muscle performances.

More severe are perhaps issues of storage capacity in memory formation that are not considered here. The Kenyon cells in the hexapod mushroom bodies are among the smallest (nerve) cells in arthropods probably for this very reason. Smaller cells provide more capacity for the particular cellular function, here storage capacity through modification of synaptic contacts. As outlined above, this option does not exist for muscle cells due to the necessary accommodation of contractile machinery, and it is restricted for motoneurons due to the reduced conduction velocity with small axon diameters, among other reasons.

**Fig. 2:**
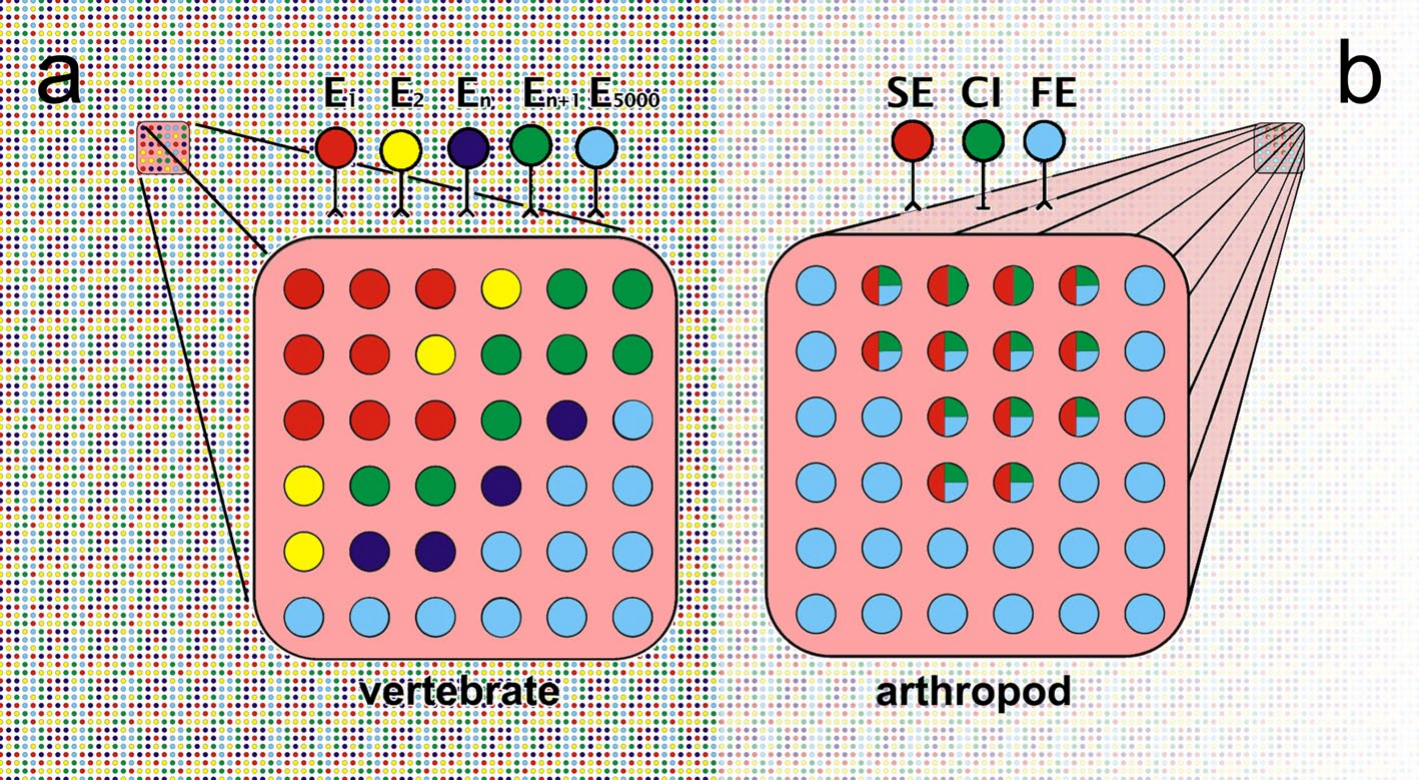
Comparison of vertebrate and arthropod muscle—cell numbers and innervation patterns. A fibre bundle of a *vertebrate muscle* is illustrated schematically in **a**. The fibre bundle is indicated to be the enlarged part of a much larger muscle; about 16,000 fibres of that muscle form the background of the figure, corresponding to a medium-sized muscle in a small mammal like the cat. Muscle features relevant in the present context are: (1) the whole muscle consists of several thousand muscle fibres, indicated by the figure background; (2) each muscle fibre is supplied by only one motoneuron, indicated by matching colours of motoneurons indicated *above* the fibre bundle and muscle fibres in the bundle; (3) the muscle is supplied by several hundreds to thousands of motoneurons, indicated by index numbers of the motoneurons. A whole *arthropod* leg *muscle* is indicated schematically in **b**. The corresponding muscle features are: (1) the whole muscle consists of a few tens (here 36) to a few hundreds of muscle fibres; (2) each muscle fibre is supplied by one or several, sometimes all motoneurons, again indicated by matching colours between motoneurons and (sections of) muscle fibres; this means that motor units usually overlap, as is apparent from the mixed colour fibres; (3) the muscle is supplied by few, often just three, motoneurons; these are typically one or a few fast motoneurons (*blue*), one or a few slow motoneurons (*red*), and one common inhibitory motoneuron (*green*) (see also Fig. 3, where intermediate muscle fibres are shown; Fig. 6)

**Fig. 3:**
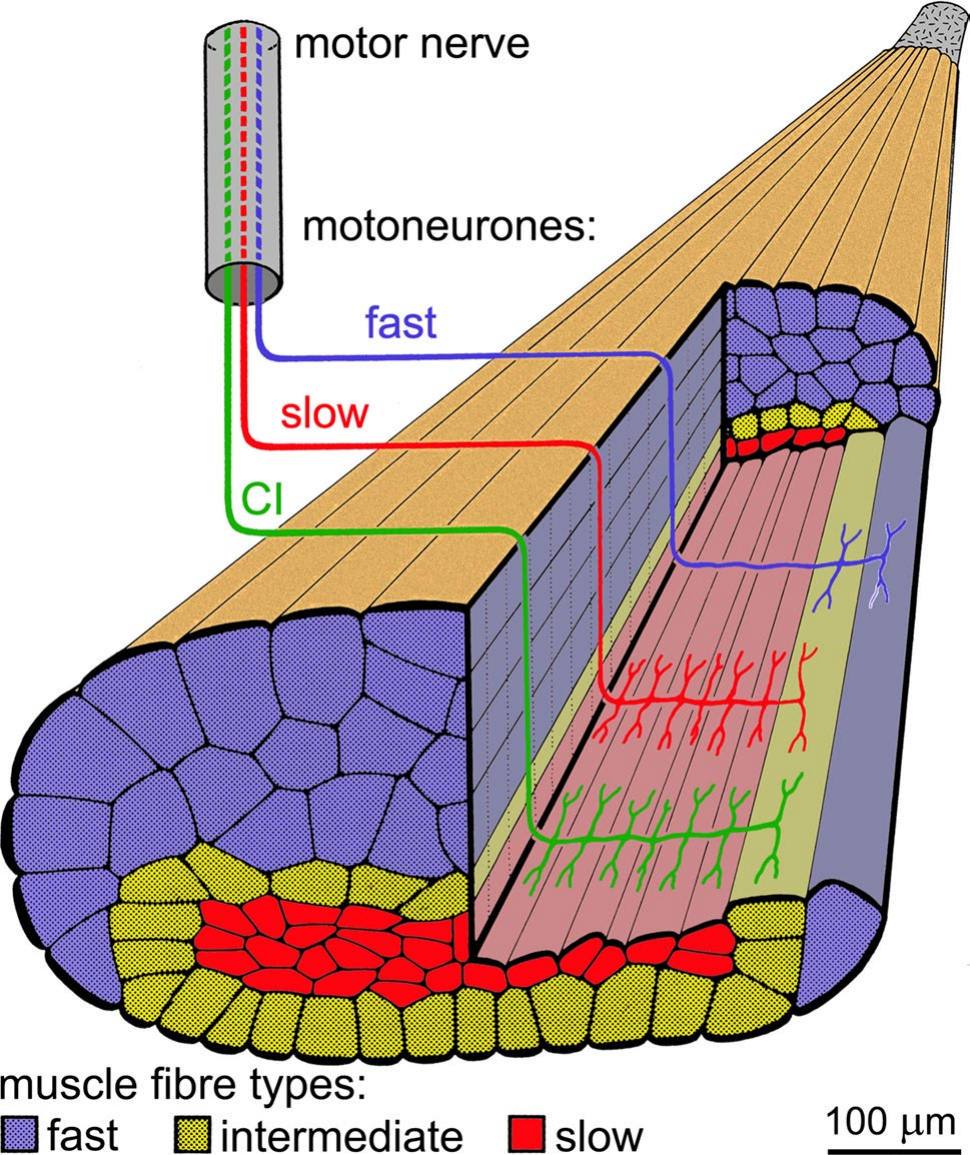
Arthropod muscle—fibre composition and innervation pattern. The different muscle fibre types are distinguished by colour coding, as is their motoneuron supply. Note the strictly parallel innervation of muscle fibres by the slow and CI motoneurons. Semi-schematic drawing of a locust leg muscle (M92) with a portion of the muscle cut away to illustrate anatomy and innervation patterns; adapted from (Müller et al. 1992). This muscle consists of roughly equal numbers of slow (30), intermediate (27) and fast (34) fibres. Diameters increase from slow to fast fibres, in parallel to changes in contractile and metabolic properties. Innervation of a given muscle fibre by more than one motoneuron, particularly common for intermediate type fibres, results in overlap of motor units

The muscles of mammals contain between a few hundred and a few million muscle fibres, depending on animal and muscle size (Rehfeldt et al. 1999). In larger animals, muscle fibres may be increased in length by the fusion of several longitudinally arranged primordial muscle cells during development, producing a syncytial muscle fibre with several nuclei. In the maggot of the fruit fly some body wall muscles are represented by just a single muscle fibre. Locust and crab muscles—as two important and particularly well-studied model systems in arthropod neurobiology—occupy intermediate ranges of fibre numbers. Leg muscles in these two comparatively large arthropods may contain from fewer than 30 to just above 500 fibres (e.g., Rathmayer and Maier 1987; Müller et al. 1992). The situation is similar for the numbers of motoneurons supplying these muscles. Most notably, malacostracan and hexapod leg muscles are typically innervated by just three to five motor axons, including one inhibitory motoneuron. Extreme numbers range from 1 to 19 motoneurons per muscle (Figs. 2, 3, 6).

**Fig. 4:**
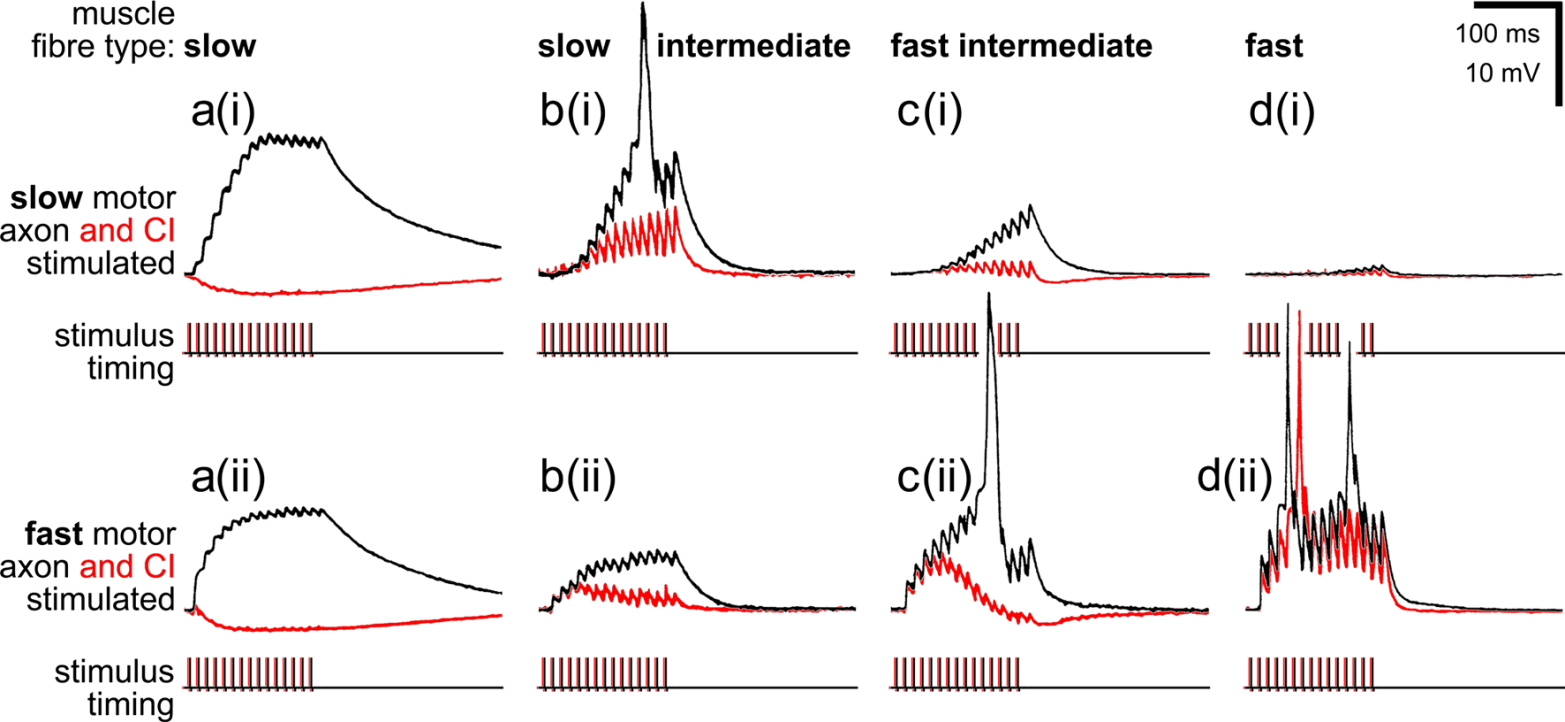
Heterogeneity of muscle fibre properties in arthropod motor units, exemplified in crab muscle recordings. Intracellular muscle fibre recordings illustrate electric properties (*top* pairs of *black* and *red* traces in **a**–**d**) in response to excitatory motor axon stimulation (*bottom* pairs of traces). Superimposed are traces recorded without (*black*) and with (*red*) parallel stimulation of the common inhibitory motoneuron (CI). Four sample muscle fibres were recorded, belonging to the “slow” [or tonic (**a**)], slow intermediate (**b**), fast intermediate (**c**) and fast [or phasic (**d**)] categories. The slow motor axon supplying the four muscle fibres was stimulated in the *upper* samples [**a**(*i*)**–d**(*i*)], and the fast motor axon in the *lower* samples [**a**(*ii*)–**d**(*ii*)]. Note that the CI motoneuron had a strong inhibitory effect on the slow muscle fibre, inhibition decreasing with fibre type towards an almost negligible effect in the fast muscle fibre. Further, the relative input strengths of the slow and fast motor axons relate to muscle fibre type. Based on recordings from the bender muscle of a walking leg in the crab *Eriphia spinifrons* (Wiens et al. 1988). Note interruption of the (otherwise always identical) stimulus traces in **c**(*i*) and **d**(*i*) to accommodate large potentials in **c**(*ii*) and **d**(*ii*) while conserving space

Each of these motoneurons supplies a subset of fibres in the respective muscle, and sometimes all muscle fibres (Rathmayer and Bévengut 1986). Different from the situation in vertebrates, the muscle fibre populations supplied by different motoneurons—the motor units—may overlap substantially (Figs. 2, 3). A given muscle fibre may thus receive input from two or three motoneurons, usually fast, slow and intermediate types. These overlapping motor units are one way to produce different mechanical performances with few control elements since a given motoneuron gains access to a larger number of muscle fibres that may have different contractile properties. Moreover, a given muscle fibre will show different electric and contractile responses to inputs from the different supply motoneurons [Fig. 4; compare sample recordings labelled (i) and (ii), respectively] (Müller et al. 1992).

Slow motoneurons have smaller diameters, in comparison to fast motoneurons, they thus have slower conduction velocities, and their synapses on the muscle fibres elicit comparatively smaller junction potentials (Fig. 4) (most arthropod muscles fibres do not generate action potentials; one notable exception are flight muscles in hexapods). These junction potentials often show pronounced summation and facilitation (Rathmayer and Hammelsbeck 1985) and they have long rise and decay time constants, allowing for maintained fibre contraction at very low motoneuron discharge rates (Rathmayer and Erxleben 1983; review in Rathmayer 1990, 1997). Fibre tonus can thus be finely graded with motoneuron discharge frequency. These properties are most pronounced in the most slowly contracting muscle fibres supplied by a slow motoneuron (Fig. 4a). The intermediate fibres typically exhibit less summation and facilitation (Fig. 4b, c) and they have higher excitation–contraction coupling thresholds, resulting in recruitment at correspondingly higher motoneuron discharge frequencies (Rathmayer and Erxleben 1983; Rathmayer and Hammelsbeck 1985; reviews in Rathmayer 1990, 1997).

The situation for fast motoneurons is complementary to the slow motoneurons. They typically occupy the other end of the spectrum of muscle fibre properties, exhibiting large axon diameters and fast conduction velocities, supplying predominantly fast contracting muscle fibres and generating large junction potentials with short rise and decay time constants (Fig. 4d). These properties typically result in twitch contractions in response to single motoneuron spikes or high-frequency spike bursts. The twitch contractions may fuse into tetanic contractions like in vertebrate muscle. Specialised fast contracting arthropod muscle such as orthopteran hexapod flight muscle may generate action potentials and exhibit twitch contractions exclusively (Josephson 1993).

Intermediate motoneurons are a broadly defined class of neurons that exhibit properties intermediate between slow and fast motoneurons and they supply muscle fibres in a correspondingly intermediate spectrum, usually including fibres also innervated by fast and slow motoneurons (e.g., Müller et al. 1992). Notably, intermediate muscle fibres are often supplied by both slow and fast motoneurons, even if no intermediate motoneuron exists for the given muscle (Figs. 3, 4).

## Fibre heterogeneity within motor units is a key feature for the smoothly graded control of muscle force

Another important difference between arthropods and vertebrates regarding neuromuscular organisation is evident from the above description. The heterogeneity of the muscle fibre population supplied by a given motoneuron, that is, of a given motor unit, has important consequences for fibre activation. This heterogeneity is the basis for a dynamic physiological recruitment of the different fibres in the motor unit. Fibre heterogeneity not only concerns junction potential size, facilitation and summation properties (Fig. 4) but also excitation–contraction coupling threshold. The fibres of a unit will thus be recruited more or less one after the other with increasing motor spike frequencies (except perhaps at the fast end of the spectrum, where bulk recruitment in fight-or-flight situations may occur). Muscle fibres innervated by more than one motoneuron will respond differently to input from these two nerve cells [Fig. 4; compare recordings labelled (i) and (ii)]. In essence, fibre heterogeneity and overlap of motor units allows gradual recruitment of the small number of muscle fibres in a muscle, occasionally even the selective activation of individual muscle fibres. Considering the graded control of an individual fibre’s contraction outlined above, a correspondingly smooth control of force output results. The dynamic range of muscle forces is thus indeed comparable to that of vertebrate muscles (reviews in Rathmayer 1990; Müller et al. 1992) with their much larger number of motor units that can be addressed individually according to the required force output.

It is almost self-evident that virtually all other muscle fibre properties, in addition to the electric properties named above, may vary considerably within a given motor unit (Rathmayer and Erxleben 1983; Rathmayer and Hammelsbeck 1985; Maier et al. 1986; Rathmayer and Maier 1987; Müller et al. 1992; reviews Rathmayer 1990, 1997). Most obvious is fibre diameter that is typically largest for fast contracting fibres and smallest for slow contracting ones (Fig. 3). This makes good sense in view of an animal’s need for these fibre types. Fast motor units will usually be activated in situations where fast contractions and large forces are required, such as predatory strikes or evasive behaviours. Slow muscle fibres, by contrast, are used in “everyday tasks” such as posture control. This does not require large forces but maintained tonic contractions and metabolic economy. In keeping with these requirements, slow contracting muscle fibres usually exhibit aerobic metabolism and may possess generous energy stores such as glycogen, while fast fibres are often anaerobic, at least to a major extent, have few energy stores and thus show pronounced fatigue. Unsurprisingly, this closely resembles the situation in vertebrates, where similar division of labour exists between different motor units. Henneman’s size principle (Henneman 1957, 1985) developed for vertebrates reflects these generally applicable rules. Briefly, Henneman’s size principle states that with increasing load, motor units are recruited starting with the smallest and gradually proceeding to the largest. This implies that slow, low-force, fatigue-resistant muscle fibres supplied by small-diameter motoneurons are activated before the fast, high-force, less fatigue-resistant muscle fibres supplied by large-diameter motoneurons.

In arthropods, the spectrum of possible muscle fibre properties is broader than in vertebrates, in addition to the above features. The spectrum includes many fibre types not discussed here, including extremely fast, stretch-activated flight muscle in dipteran hexapods (Pringle 1978; reviews in Josephson 1993; Hooper et al. 2008) that may support wing stroke frequencies beyond 500 Hz, or muscles with catch-like properties that allow the maintenance of muscle force without maintained nervous input (Hoyle 1983, 1984; Hoyle and Field 1983; review in Hooper et al. 2008), a very energy-efficient type of muscle used for long-lasting postural tasks (Rathmayer and Maier 1987).

## Peripheral inhibition is an essential element of the motor control strategy in arthropods

The structure of arthropod muscle outlined above has one major and inevitable disadvantage, outlined in the following. When fast and forceful contractions of leg or body wall muscles are required, one would expect that virtually all fibres within the muscle are activated. There are two reasons for this. First, fast and slow motor axons supplying a given muscle are usually tightly coupled in their activities. This is clearly seen in intracellular recordings where slow and fast motoneurons often show almost identical depolarisation patterns, except that in the fast motoneuron the input is distinctly smaller, reaching threshold only with stronger synaptic input (e.g., Gabriel et al. 2003; see also Henneman 1985). A slow motoneuron controls the muscle under most normal conditions with subtle variations in spike frequency. A fast motoneuron discharges spikes only at higher levels of behavioural excitation, for instance, during fast walking or running, when the slow axon is already active with high discharge frequencies. That is, in situations where the fast motoneuron spikes, the slow muscle fibres have already been activated by the slow motoneuron. Second, even if the fast axon should fire just on its own many intermediate and slow muscle fibres will be activated due to the overlap of motor units. That is, the fast axon typically supplies not just all fast but also many or all intermediate muscle fibres and several of the slow fibres (Figs. 3, 4). In some muscles, the fast axon indeed supplies all muscle fibres (Cooke and Macmillan 1983; Rathmayer and Bévengut 1986). Differential recruitment of fast and slow muscle fibres is rarely observed but occurs in particular behavioural context, such as aimed scratching movements (Page et al. 2008).

Slow muscle fibres may have slow contraction and relaxation kinetics indeed, with time constants ranging from several tens of milliseconds to well over 100 ms (Atwood et al. 1967; review in Rathmayer 1990). This illustrates that already step cycle periods in the range of 10 Hz may lead to the build-up of residual muscle tension in the walking limb, due to a continuous activation of the slowest muscle fibres. The resulting decrease in movement speed and amplitude would be detrimental not just in life threatening situations where a fast escape is mandatory.

**Fig. 5:**
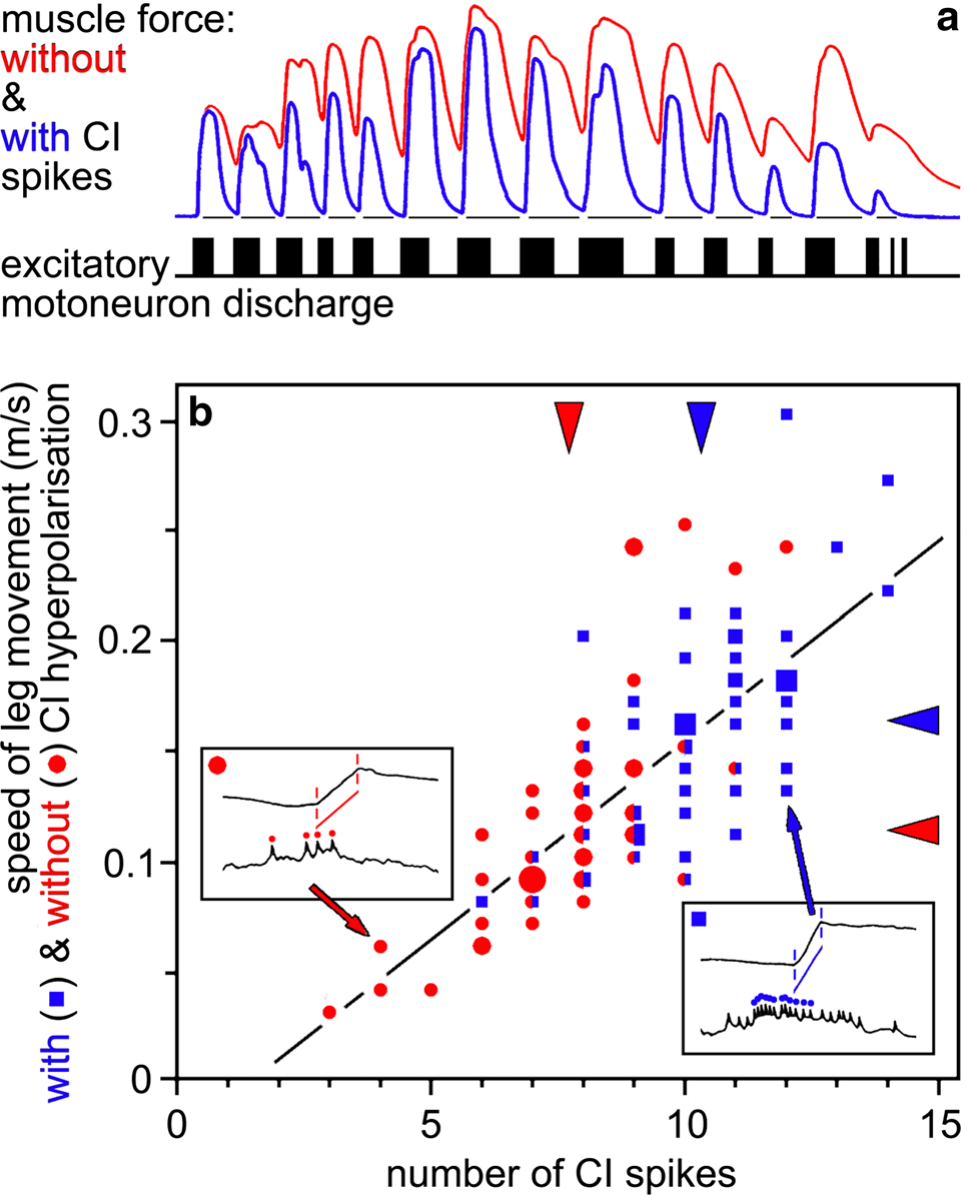
Impact of common inhibitory motoneurons on muscle contraction (**a)** and leg movement (**b)**. **a** Force produced by a crab leg muscle (*top* two traces; dactylopodite closer muscle; see Fig. 6) in response to stimulation with a pattern of excitatory (slow) motoneuron discharge (*bottom* trace) recorded previously during walking. The recorded CI discharge was selectively omitted (*red*) or applied (*blue*) (trace of tonic discharge not shown) during slow motoneuron stimulation. Note complete relaxation in between contraction peaks with CI discharge present, and build-up of residual muscle force, up to about 40 % of peak values, without CI spikes. Maximum peak force was about 80 mN. **b** In a tethered locust walking in a treadwheel, the speed of middle leg protraction during swing was measured at mid tibia level, and is plotted on the ordinate. CI1 discharge was recorded intracellularly and spike numbers of the burst associated with the swing are plotted on the abscissa. The two insets illustrate the measured parameters, slope of the swing (upstroke in leg position recording), and spike numbers in the CI recording; two data points with corresponding values are indicated (although the samples are from a different animal). *Blue square* data points were recorded during normal walking, CI was hyperpolarised to reduce spike discharge during the recording of *red circle* data points. *Arrowheads* opposite ordinate and *abscissa* indicate corresponding mean values of movement speeds and spike numbers, respectively. Note that experimental reduction of CI spike discharge also reduced leg movement speed. (**a**) Courtesy of Werner Rathmayer (recording from a set of experiments reported in (Ballantyne and Rathmayer 1981); (**b**) adapted from (Wolf 1990)

**Fig. 6:**
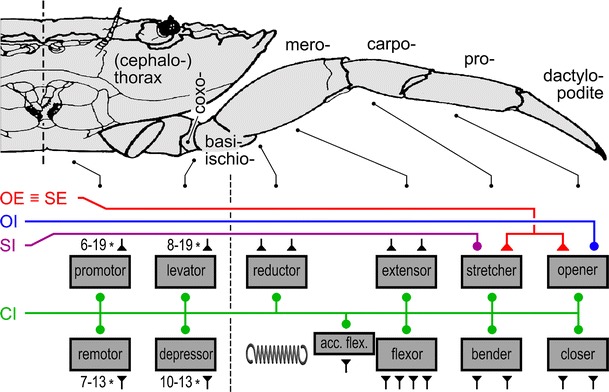
Three inhibitory motoneurons supply the decapod crustacean leg muscles. Thorax and walking leg of a crab are outlined at the *top*, with leg articles labelled. The corresponding sets of antagonistic muscles are indicated as *boxes* at the *bottom* (dactylopodite contains no muscles; *spring symbol* indicates elastic cuticle elements that work against the reductor of the meropodite, instead of an absent meropodite promotor). Inhibitory motoneuron supply is indicated in colours (*green*, common inhibitor; *violet*, stretcher inhibitor; *blue*, opener inhibitor; inhibitory synapses, *dots*). The common excitor of stretcher and opener muscles is drawn in *red* (excitatory synapses, *triangles*). Other excitatory motoneurons are indicated as *black* synapses; motoneuron numbers above four are given as numbers; number ranges indicate variation between examined species [based on (Faulkes and Paul 1997)]. *Dashed lines* indicate carapace midline at *top*, and leg autotomy plane level of leg article fused from basi- and ischiopodites

This is the context where inhibitory muscle innervation becomes essential. The common inhibitory motoneurons innervate the fibres of a given muscle strictly in parallel to the slow motoneuron(s) (Rathmayer and Erxleben 1983; see also Atwood et al. 1967) (Fig. 3). That makes functional sense considering the fact that CIs inhibit the slow muscle fibres during fast movements, while there is no need for the inhibition of fast contracting muscle fibres (exceptions exist for specific inhibitory motoneurons, below). In this way, the build-up of residual tension is avoided and cyclic fast movements can proceed without antagonistic sets of muscles impeding each others’ contractions. The inhibition is as finely tuned to the required movement velocity as has been described above for the subtle control of slow muscle fibre activation. CI spike frequency determines, among other parameters, membrane time constant and thus the summation of inhibitory junction potentials and the inhibition of a given muscle fibre’s recruitment. The inhibitory supply of slow muscle fibres is as varied as the excitatory supply, with the most slowly contracting fibres receiving the strongest inhibition (Ballantyne and Rathmayer 1981; Worden and Camacho 2006; review in Rathmayer 1990). Again, this is what should be expected from a functional perspective. The slower the contraction and relaxation kinetics of a given muscle fibre are, the slower is the speed of antagonistic muscle contraction at which inhibition needs to take effect, that is, the more effective inhibition by CIs needs to be already at low spike discharge rates. With higher movement speeds, CIs will discharge more vigorously and inhibit, also, the muscle fibres with intermediate contraction kinetics, allowing faster movement cycles. In other words, CI discharge rate adjusts the performance of a muscle to the movement speed required for a given motor task (Fig. 5). This function of CI neurons is the same for all the muscles involved in a given movement of body or appendages. That in turn resolves the initially intriguing observation that a single common inhibitory motoneuron supplies all muscles of a walking leg in crabs (Rathmayer and Bévengut 1986), and many other malacostracans (e.g., Cooke and Macmillan 1983) (Fig. 6). This CI serves the global adjustment of movement speed in all muscles of the leg according to the momentary behavioural context.

It is not just inhibition of slow muscle fibres, in the sense of preventing their activation that promotes fast muscle performance. The inhibitory postsynaptic junction potentials are produced by the opening of membrane channels for potassium or chloride ions, and these reduce membrane resistance and thus membrane time constant (Atwood et al. 1967). The reduced membrane time constant accelerates the relaxation of muscle fibres, an effect that is certainly relevant at least for intermediate muscle fibres that are not completely inhibited during movement (Ballantyne and Rathmayer 1981). The cellular mechanisms of inhibition are briefly discussed below.

In summary, inhibition through CI neurons of muscle fibres that are supplied by slow excitatory motoneurons serves the adjustment of muscle performance to behavioural requirements in the velocity domain (review in Rathmayer 1990). During active movement, spike frequency is typically increased in all motoneurons, fast, slow, and inhibitory. This leads to the recruitment of fast muscle fibres and to increased input to slow and intermediate muscle fibres. At the same time, spike discharge in the CIs inhibits slow and intermediate muscle fibres, leading to the inactivation of the slowly contracting fibres and an accelerated relaxation of intermediate fibres, and perhaps their decreased activation. The inhibitory effects are mediated via both pre- and postsynaptic mechanisms (below).

## Common inhibitors control muscle relaxation and movement speed

The function of CI neurons outlined above is nicely demonstrated in nerve-muscle preparations (Ballantyne and Rathmayer 1981) and, most convincingly, in tethered walking (Wolf 1990) and scratching (Calas-List et al. 2014) animals. CI discharges were experimentally altered in these studies while leaving the excitatory input unchanged (Fig. 5), thus allowing direct observation of the effects of CI activity on muscle force and behaviour. Two of these illustrative experiments shall be summarised in the following (Fig. 5). It is important to note in this context that in hexapods, the three common inhibitory motoneurons that supply the leg muscles apparently fulfil the same basic function. By contrast, only one common inhibitor supplies all leg muscles in malacostracan crustaceans. The two remaining inhibitors have a different function that is discussed below.

In the first example, the discharge patterns of the three motoneurons supplying a leg muscle, the dactylopodite closer, were recorded with implanted electrodes in a walking crab (Ballantyne and Rathmayer 1981). The recorded patterns were used to stimulate the respective motor axons in a nerve-muscle preparation (Fig. 5a). Applying just the excitatory input produced clear force peaks in the step cycle. However, the muscle did not relax completely between those force peaks. On the contrary, residual tension was in the range of 40 % of maximum force output. Adding the respective CI spikes to the stimulation regimen resulted in complete muscle relaxation between force peaks. During these times, the antagonistic muscle would be free to move the respective leg joint in the opposite direction to control alternating limb movements in walking with full amplitude.

In the second example, the ventral nerve cord of a locust walking on a treadwheel was accessed through a small opening in the ventral cuticle, and CI1 soma was impaled with a microelectrode (Wolf 1990). This allowed current injection into CI1 to alter the spike discharge of the inhibitory motoneuron (Fig. 5b). Reducing the spike discharge by hyperpolarizing current injection reduced the speed of leg movement during the swing phase of the step cycle. This is a direct behavioural proof for the global function of CI1 neuron in adjusting muscle performance to higher movement speeds.

CI spike activity was recorded during normal walking behaviour in these and other studies (Ballantyne and Rathmayer 1981; Burns and Usherwood 1979; Wolf 1990). These results agree closely with what would be expected on the basis of CI function outlined above. When the animal, whether crab or locust, maintains its posture during rest, CI neurons are not active at all. As soon as the animal starts walking, CI spikes appear and discharge frequency increases with increasing locomotor speed. Such increased CI discharge will inhibit most strongly the slowest muscle fibres and also affect intermediate ones and thus facilitate faster movement. There is also modulation of CI spike frequency in the step cycle. Spike frequency peaks just before the start of the swing phase, when the leg is moved briskly through the air to return to its anterior reversal point where it is put on the ground and can again support and propel the animal. This peak just before the fast phase of the leg movement makes good sense not just for adjusting the swing phase muscles to the appropriate speed but also to fully relax the antagonistic stance phase muscles. Intriguingly, CI starts to depolarise, although not necessarily to spike, well before movement commences, apparently in the context of preparing the whole motor system for the impending start of action (Wolf 1990, 1995).

In a recent study, Calas-List and coworkers (Calas-List et al. 2014) altered CI discharge in a similar manner, or they abolished inhibitory input to the leg muscles completely. The latter was achieved by filling the CI with dye during intracellular recording and later killing it by laser photoablation. Reducing CI discharge significantly reduced the speed of aimed scratching movements in keeping with the results of the above studies, and increasing CI discharge increased movement speed. Unexpectedly, these imposed changes in movement speed did not compromise accuracy of the scratching movements. This is in contrast to common motor control strategies in vertebrates, where higher accuracy of movement is often achieved through increased joint stiffness and accordingly slower movements (Selen et al. 2009; Wong et al. 2009).

CI neurons receive input not just from pattern generating circuitry that controls walking behaviour (Rykebusch and Laurent 1993) but from sensory organs as well, primarily mechanoreceptors (Fourtner and Drewes 1977; Ritzmann and Camhi 1978; Cattaert et al. 1993; Schmidt and Rathmayer 1993; Matheson and Field 1995). This direct, sometimes monosynaptic, input presumably serves the preparation of the animal for fast evasive movements in response to disturbances. There are no detailed studies on the behavioural role of the sensory inputs to CI neurons as yet, however.

## Body wall inhibitors

Inhibitory motoneurons supplying leg muscles in malacostracans and hexapods have been studied in quite some detail. A group of inhibitory motoneurons that innervates the body wall muscles has received much less attention, by comparison. It is clear nevertheless that not just the leg muscles used in walking but many muscles of the body wall as well are supplied by inhibitory motoneurons (Atwood et al. 1967; Msghina and Atwood 1997; Murchinson and Larimer 1997; Drummond and Macmillan 1998; Schmäh and Wolf 2003). Notably, these are also common inhibitors, with few exceptions (Bräunig et al. 2006). The common innervation pattern suggests that these body wall inhibitors serve a similar global function in the adjustment of movement speed as do the leg common inhibitors. Not all body wall muscles are supplied by inhibitors, however, and physiological experiments examining this hypothesis have not been carried out.

Occasionally, even the muscles of appendages may not be supplied by (common) inhibitory innervation. This is true for the scaphognathite, an appendage that is used in malacostracans to ventilate the respiratory cavity below the carapace (Moody-Corbett and Pasztor 1980). In this function, the scaphognathite performs constant slow ventilator strokes. Different from the leg muscles, thus, the spectrum of motor performance is narrow indeed, consisting of muscle contractions and relaxations with essentially constant and low force and speed values. This corroborates the above notion that inhibitory innervation serves the division of labour in heterogeneously composed motor units to cover a broad spectrum of force and particularly speed performances. A lack of inhibitory innervation may thus be indicative of a constant demand on muscle performance; in the case of body wall muscles, perhaps ventilation. Similarly notable is the absence of inhibitory motoneuron supply for stomach muscles that are concerned with constant rhythmic chewing movements.

## Cellular mechanisms of muscle inhibition

Inhibitory motoneurons have been a favourable and very successful model system for studying the cellular mechanisms of inhibition. Major reasons are the good experimental accessibility of the arthropod neuromuscular junction, in malacostracan crustaceans in particular, and the small number of motor units and their reliable identification (Atwood and Tse 1993; Clarac and Pearlstein 2007). Mechanisms of inhibition are shared among excitable cells and thus mostly apply not just to the neuromuscular junction but to neuro-neuronal inhibitory synapses as well, and many aspects extrapolate to other animal groups, including vertebrates. The cellular mechanisms of inhibition have been reviewed repeatedly and in detail elsewhere (for arthropods see Nicoll and Alger 1979; Atwood and Tse 1993; Clarac and Cattaert 1996), as has the study of muscle inhibition in arthropods (Atwood 1973; Wiens 1989; Rathmayer 1990; Atwood and Tse 1993; Clarac and Cattaert 1996). A brief summary focussing on aspects relevant for arthropod inhibitory motoneurons shall suffice, therefore.

**Fig. 7:**
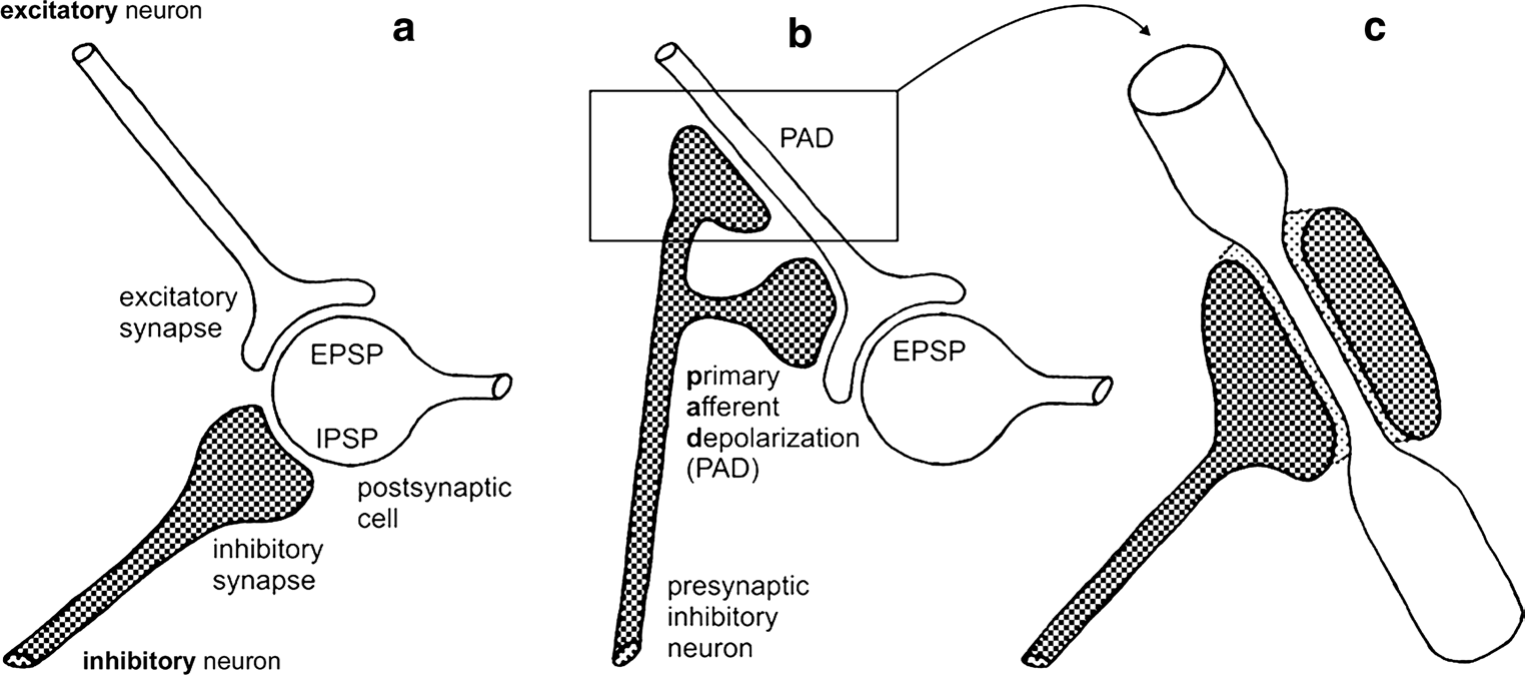
Pre- and postsynaptic inhibition in comparison; inhibitory neurons are shaded. Postsynaptic inhibition is shown in the form of a standard diagram in **a**. Presynaptic inhibition (**b** and **c**) may impinge either on the excitatory synaptic bouton [*lower* inhibitory synapse (**b**)] or on the terminal axon branches [*top* synapse (**b**, **c**)] via shunting inhibition. Note tapered axon segment enveloped by inhibitory synaptic contact in **c**

Inhibitory motoneurons bring about their inhibition of muscle excitation by both post- and presynaptic mechanisms (Fig. 7). Both mechanisms use gamma-aminobutyric acid (abbreviation GABA) as neurotransmitter. GABA was identified as an inhibitory transmitter at the crustacean neuromuscular junction (Otsuka et al. 1966) soon after its discovery as a “neuronal secretion” (Florey 1954; Bazemore et al. 1957). The GABA receptor proteins located in the subsynaptic membrane have been characterised into three types, an ionotropic GABA-a receptor and metabotropic GABA-b and GABA-c receptors (Dudel and Hatt 1976; Jackel et al. 1994; Fischer and Parnas 1996a, b; Pearlstein et al. 1997; Rathmayer and Djokai 2000). These different types of transmitter receptors appear to be specifically associated with the pre- or postsynaptic membranes (Miwa et al. 1991). Study of the GABA receptor types in arthropods is impeded by the fact that readily available receptor agonists and antagonists that have been characterised in vertebrate systems do not necessarily work in exactly the same way for arthropod transmitter receptors (Jackel et al. 1994; Pearlstein et al. 1997). This is probably due to the long evolutionary times of divergence that are reflected in correspondingly large differences in amino acid sequences (Hille 1984).

*Postsynaptic inhibition* of muscle fibres works in the same way as postsynaptic inhibition among neurons, through the opening of channels for potassium (K^+^) or chloride (Cl^−^) ions (Fuchs and Getting 1980; Miwa et al. 1991; Jackel et al. 1994; Pearlstein et al. 1997). Since the equilibrium potential for these ions is typically close to −70 mV, opening of the ion channels counteracts any depolarisation of the muscle cell towards excitation–contraction coupling threshold. This threshold is more positive, depending on muscle fibre type starting at about −60 mV (Huddart and Abram 1969; Fischer and Florey 1983). Inhibitory and excitatory inputs to the muscle fibre thus interact through their respective synaptic currents on the membrane of the muscle cell. As noted above, arthropod muscles typically do not produce spikes but rather exhibit graded contractions that depend on the momentary membrane potential of the cell. Inhibitory input will thus reduce any contractile response but not necessarily abolish it completely. The synaptic boutons of excitatory and inhibitory neuromuscular junctions are usually closely apposed to each other and run much of the length of a muscle fibre (Tse et al. 1991; Msghina and Atwood 1997). Without action potentials, depolarising the whole muscle fibre membrane by synaptic contacts along the fibre length via polyterminal innervation is indeed essential (polyterminal innervation is not indicated in Fig. 3 to avoid crowding). The close proximity of excitatory and inhibitory inputs certainly favours local interaction of excitatory and inhibitory junction currents. It may lend some dominance to inhibitory input where the junction contacts are larger and presumably equipped with more ion channels for the inhibitory motoneuron (Walrond et al. 1990). At the same time, the ion channels opened by GABA reduce membrane resistance and thus accelerate repolarisation after excitatory transmitter release has terminated (Atwood et al. 1967), as noted above. In summary, postsynaptic inhibitory input will reduce the amplitude of muscle fibre contractions and accelerate their subsequent relaxation.

The mechanisms of *presynaptic inhibition*, too, are similar between neuromuscular junction and nerve cell synapse. Presynaptic inhibition reduces transmitter release from the synaptic terminal it impinges upon. This is again achieved by the opening of channels for K^+^ or Cl^−^ ions (reviews in Atwood and Tse 1993; Clarac and Cattaert 1996). However, there are no major depolarising ion conductances that could be reduced in amplitude in the axon terminals. Rather passive action potential propagation in the terminal axon branches is decreased or eliminated. This occurs by shunting inhibition, that is, the conductances for K^+^ or Cl^−^ ions reduce membrane resistance which leads to a reduced length constant and thus decreases or abolishes the voltage change that eventually arrives at the synaptic terminal to elicit transmitter release (Dudel and Hatt 1976; Smith 1978; Baxter and Bittner 1991). Alternatively, if the action potential is still propagated actively by voltage-gated channels for sodium ions, the K^+^ or Cl^−^ conductances reduce spike amplitude below the threshold for further spike propagation (Atwood and Tse 1988). Such presynaptic inhibition is common not only in peripheral inhibition through inhibitory motoneurons but ubiquitous throughout the nervous system, including presynaptic inhibition of sensory inputs (Cattaert et al. 1992; Burrows and Matheson 1994; for vertebrates see Rudomin 1990).

In some neurons, the equilibrium potential for Cl^−^ ions is slightly more positive than resting membrane potential, say, −65 mV rather than −80 mV. If chloride channels are opened by an inhibitory transmitter like GABA, the membrane potential thus slightly depolarises, rather than hyperpolarises. The effect is nonetheless an inhibitory one since the membrane potential is clamped by the chloride conductances to a value well below the threshold for transmitter release, to −65 mV in this example. Such a depolarising (presynaptic) inhibitory input is seemingly paradox, which has made an understanding of presynaptic inhibition more difficult initially.

Like in the case of postsynaptic inhibition, the structure of the synaptic contact may contribute significantly to the inhibitory effect (Jahromi and Atwood 1974). A small presynaptic terminal on an axon terminal arborisation with relatively large diameter will have little effect in reducing passive spike propagation and probably will not influence active spike propagation at all. By contrast, a large synaptic contact that actually envelopes a very thin terminal axon branch will represent a sink for any depolarising currents and thus eliminate transmitter release in the adjacent terminal (Atwood et al. 1984) (Fig. 7c).

Arthropod muscles do not only respond to neurotransmitters but often to neuromodulatory inputs as well. Octopamine (Pflüger and Stevenson 2005) and proctolin (Allgäuer and Honegger 1993) are prominent examples here. Neuromodulation may change membrane resistance and thus integration properties of the muscle fibre membrane, or it may depolarise the cell. In this way, neuromodulatory input will interact with inhibitory input discussed above, particularly with postsynaptic inhibition. While neuromodulatory input will change the excitability, force output and recruitment pattern of the muscle in question, the functional principles of (common) inhibitory muscle innervation remain unaffected, setting muscle performance to higher speeds of repetitive movement cycles. Any detailed study of the interaction of neuromodulatory and inhibitory input to arthropod muscle is missing to date, however.

## Limb inhibitors in pterygote hexapods, decapod crustaceans, and scorpions share ancestral traits, suggesting homologies

The above outline of common inhibitory motoneuron function has dealt with orthopteran hexapods and malacostracans almost exclusively, both representatives of the Mandibulata. This was inevitable since these two arthropod groups have served as the experimental systems for the investigation of virtually all aspects of peripheral inhibition (reviews in Wiens 1989; Atwood and Tse 1993; Rathmayer 1997; Clarac and Pearlstein 2007). The fact that neuromuscular organisation is very similar in all arthropod groups suggests that possession of inhibitory motoneurons is a general arthropod feature. In particular, the main arguments for the necessity of inhibitory control of muscle fibre recruitment are valid for all arthropods, namely, the comparatively small numbers of muscle fibres and motoneurons available. Some intriguing data on chelicerates indeed exist, and more tentative results on chilopods, verifying the basic assumption of the existence of inhibitory motoneurons throughout the arthropods, and warranting comparison from an evolutionary perspective. The comparison below relies on the outline of the complement of inhibitory motoneurons supplying the leg muscles of malacostracans and hexapods provided above, just after the Introduction. The probable homologies of malacostracan CI and hexapod CI1, and of malacostracan SI and OI and hexapod CI2 and CI3, respectively, are explained in this context.

The existence of inhibitory motoneurons has been demonstrated in chelicerates, namely in scorpions (Wolf and Harzsch 2002b), and appears likely in chilopods (Harzsch et al. 2005). Inhibitory motoneuron supply of walking leg muscles would thus indeed appear to be a general and plesiomorphic feature in the arthropods. However, none of the remaining arthropod groups has as yet been examined in this respect. It would be particularly interesting, therefore, to look for inhibitors in apterygote hexapods, in the remaining myriapods such as the diplopods, in araneid chelicerates (but see Maier et al. 1987), and in onychophorans and annelids, and perhaps even in molluscs for an outgroup comparison. Holometabolous hexapods have not been examined with respect to peripheral inhibition either, a group that appears interesting with regard to evolutionary specialisation.

Inhibitory motoneurons actually lend themselves well for comparative studies since an initial identification is achieved with relative ease (technical problems in detail notwithstanding). A backfill of the main leg nerve marks the motoneurons supplying the leg muscles, and subsequent immunohistochemistry directed against GABA, the transmitter of inhibitory motoneurons, marks candidate inhibitory moto- and interneurons. Any neuron bearing a double label after this procedure is a good candidate for an inhibitory motoneuron. This is because there are no other neurons known that are motoneurons in the sense that they have an axon in a motor nerve, and that are also immunoreactive to the inhibitory transmitter GABA. This is how the candidate inhibitors in scorpions and chilopods have been identified (Fig. 1c), in the case of the scorpions with an additional electrophysiological proof of (multiple) inhibitory innervation of leg muscles and detailed neuroanatomy (Wolf and Harzsch 2002b).

Inhibitory motoneurons in scorpions and chilopods are not just present but appear in an intriguing pattern reminiscent of the situation in malacostracans and hexapods. Most notably, though, there are not just three inhibitory neurons in scorpions but three groups of neurons (Wolf and Harzsch 2002b) (Fig. 1c). These groups occupy positions that are typical of the single inhibitors in malacostracans and hexapods: one group, probably corresponding to hexapod CI1, is located just contralateral to the leg it innervates near the ganglion midline, a second group, probably corresponding to hexapod CI2, occupies a position slightly more anterior and clearly ipsilateral to the midline, and a third group, probably corresponding to hexapod CI3, is located more posteriorly and laterally, not far from the posterior nerve roots.

The fact that in scorpions there are small groups of neurons, numbering between two and about 12, instead of single inhibitors is in line with the fact that there are up to ten times as many motoneurons supplying a given leg muscle compared to malacostracans and hexapods (Wolf and Harzsch 2002a; and perhaps the situation is somewhat similar in chilopods). It is at present unclear how this situation may be interpreted. A larger number of neurons (though still very much smaller than in vertebrates) may be an ancient, plesiomorphic character that evolved towards smaller nerve cell numbers and allowed smaller body sizes in hexapods and modern crustaceans. This would appear as a likely interpretation in view of the large marine eurypterids in the fossil record (Dunlop and Webster 1999) as predecessors of extant chelicerates. Alternatively, the nerve cell numbers increased secondarily for as yet unknown reasons.

## Specific inhibitors appear to represent derived characters, evolved for particular functions

Another set of features that may represent a derived state in malacostracans are the specific inhibitors (abbreviated SIs) in crabs and crayfish (Wiens et al. 1988; Wiens 1989). In these animals’ legs, the extensor muscles of the two most distal articles share a single excitatory motoneuron. These muscles are termed the stretcher (of the propopodite) and the opener (of the dactylopodite, or terminal leg article). The stretcher excitor and the opener excitor are thus one and the same motoneuron. To uncouple extension movements in these two leg articles, the two muscles need one specific inhibitor each that prevents contraction of the muscle meant to stay relaxed whenever the common excitor is active. While a strict uncoupling of stretcher and opener may not often be necessary in walking—here the two leg articles usually work synergistically during extension movements of the whole leg—such uncoupling is essential where the dactylopodite works as a claw that is used to grasp or hold on to objects. This is particularly evident in the large claws on the first set of legs in crabs and crayfish but it is also true for the small claws on the normal walking legs of crayfish. It makes functional sense in this context that the excitatory motoneuron to one of the antagonistic muscles, the closer, firstly shares synaptic inputs with the opener inhibitor and secondly receives excitatory input from the inhibitory motoneuron itself via mutual central moto-motoneuronal synapses (Wiens and Atwood 1978; Bévengut et al. 1996; see also Pearce and Govind 1993). This apparently supports relaxation of the opener muscle while the closer muscle is activated.

A specific inhibitory motoneuron also exists in the thorax of the locust, in this case an inhibitor of an intersegmental body wall muscle connecting pro- and mesothorax (Bräunig et al. 2006). The function of this specific inhibitor is unknown. It might be an evolutionary remnant and as such indicative of a plesiomorphic complement of three body wall inhibitors per segment (Bräunig et al. 2006).

As outlined above, the two specific inhibitory motoneurons supplying the malacostracan leg are necessary to uncouple the stretcher and opener muscles innervated by a single common excitatory motoneuron. The existence of a common excitatory motoneuron is an extremely unusual situation by any standards. When considering the available data, the following tempting but still preliminary interpretation emerges on how this innervation pattern evolved (Wiens and Wolf 1993). The situation in (orthopteran) hexapods is outlined first (Fig. 1b) since it may represent the ancestral state in the present scenario, although the direction of evolutionary change is not unequivocally clear.

(i) The inhibitors in the hexapods that are presumably homologous to the malacostracan specific inhibitors are common inhibitors CI2 and CI3 (above); they supply four distal leg muscles and serve common inhibitory motor control as outlined above.
(ii) The last leg article in hexapods, is considered to be the claw for the present purpose; this article has only a flexor muscle (Radnikow and Bässler 1991) but no extensor; claw extension is by elastic forces of cuticle and tendons.
(iii) The extensor muscle in the hexapod leg that is closest to the claw is the levator of the tarsus, a muscle that is supplied by a single excitatory motoneuron.

If active extension of the last leg article in an ancestral, hexapod-like arthropod leg should have evolved, it is not surprising that the extensor muscle of the adjacent proximal leg article was recruited for the purpose. As in the walking legs of extant crabs, co-contraction of the resulting two extensor muscles of the two distal most leg articles would not have been detrimental, even though inevitable due to the existence of just a single excitatory motoneuron, today’s common excitor of stretcher and opener muscles. Gradual evolution of specific inhibition by restrictions of the two distal common inhibitors’ fields of innervation would have conveyed similarly gradual sophistication of leg motor control, eventually conferring the advantages of a dexterous independent claw movement. In parallel to a restriction of the distal, and ultimately specific, inhibitors’ innervation, the proximal common inhibitor would have extended its field of innervation to supply all leg muscles eventually. In this scenario, vestigial innervation of the dactylopodite closer muscle by the stretcher inhibitor (Wiens and Atwood 1975; Wiens 1991, 1993) may be interpreted as a “leftover” innervation from common inhibitory function of the stretcher inhibitor, and likewise vestigial innervation of the dactyl opener by the common inhibitor (Wiens 1985, 1989; Wiens and Wolf 1993) may represent an incomplete spreading of the common inhibitor to all leg muscles (Fig. 1a). As indicated above, these are at present interpretations, and the direction of evolutionary change cannot be addressed without more detailed studies of other arthropod groups and developmental processes.

## Developmental origin of inhibitory motoneurons

A study of the development of inhibitory motoneurons CI1 and CI3 has been carried out in the locust (Wolf and Lang 1994). While this study cannot provide indications towards the above question on the evolution of specific inhibitory motoneurons in malacostracans, it certainly produced interesting data with regard to the evolution of inhibitory motoneurons per se.

Developmental origin of inhibitory motoneurons was traced by incubation of cultured locust embryos with bromodeoxyuridine (abbreviation BrdU) for a brief interval. This agent labels newly synthesised genetic material in dividing cells, including neurons. In this way, immunocytochemical staining for BrdU after incubation for selected time intervals in the developing embryo allowed identification of the progeny of the different neuronal stem cells, or neuroblasts. Additional injection of the fluorescent marker Lucifer yellow into the primordial neurons was able to identify inhibitory motoneurons CI1 and CI3 as the first progeny of neuroblast 5.5. Primordial neurons stay dye-coupled for some time after they have been produced by their neuroblast, facilitating identification of groups of progeny sharing the same origin. It turned out that CI1 and CI3 are produced by a neuroblast that does not generate any other motoneurons. Instead, neuroblast 5.5 gives rise to inhibitory interneurons, probably exclusively so. That is, according to their developmental origin CI1 and CI3 are closely related to inhibitory interneurons rather than to “normal” excitatory motoneurons. Excitatory motoneurons are produced by a set of neuroblasts located at some distance from and not related to 5.5. A similar line of argument as for CI1 probably holds for CI2, although this inhibitor is produced by a different neuroblast, located in the immediate vicinity of neuroblast 5.5. Development of CI2 has not been studied in any detail.

A possible scenario for the evolutionary origin of inhibitory neurons is suggested by this developmental origin of CIs. Inhibitory motoneurons might have originated from “misdirected” inhibitory interneurons that sent their axonal growth cones into the periphery rather than to targets in the central nervous system, due to a change in the expression of cell adhesion and receptor molecules in their cell membranes, for example. This is as yet pure speculation, however, and detailed genetic and developmental analyses would be necessary to lend more substance to this idea. It is, however, intriguing under this perspective that inhibitory motoneurons in malacostracans possess interneuronal properties in the sense that they make synaptic output connections in the central ganglion. One example are the mutual synapses between the dactylopodite closer excitors and the opener inhibitor, as mentioned above (Wiens and Atwood 1978). Such synapses are rare in hexapods (e.g., Burrows 1973) but rather common in malacostracans (above; see also Pearce and Govind 1993; Pearlstein et al. 1998) and also in other protostomes, such as leeches.

## Outlook

Inhibitory motoneurons are not just an integral part of arthropod motor control strategy but warrant further study with regard to several aspects, ranging from cellular and molecular mechanisms of inhibition to developmental and evolutionary questions. The latter should profit from modern “evo-devo” approaches, employing inhibitory motoneurons as character sets for evolutionary and developmental analysis. Of particular interest in this respect are the possible presence and the detailed structure of inhibitory motoneurons in basal, apterygote hexapod groups and in advanced, holometabolous hexapod groups. By the same token as yet little studied arthropods such as diplopods and araneids merit investigation, as well as phylogenetically more distantly related animals such as onychophorans, annelids and molluscs. Study of these topics would also help to resolve evolutionary important questions such as the origin of the comparatively large neuron numbers in the chelicerates, whether it is ancestral or derived. Although the function of common and specific inhibitors in motor control now appears to be well understood, evolution has always been good for surprises. Analysis of CI function in the hexapod antenna and particularly in hexapod body wall muscles is badly needed under this perspective. The same is true for possible interactions of peripheral inhibition and neuromodulation, both often impinging on the same muscles.

## Abbreviations

BrdU: Bromodeoxyuridine
CI: Common inhibitory motoneuron
CI1-3: Common inhibitory motoneurons 1–3 in hexapods
GABA: Gamma-aminobutyric acid transmitter
OI: Inhibitory motoneuron of the claw opener muscle in brachyurans
SI: Inhibitory motoneuron of the propodite stretcher muscle in brachyurans
